# Disruption of Endoplasmic Reticulum and ROS Production in Human Ovarian Cancer by Campesterol

**DOI:** 10.3390/antiox10030379

**Published:** 2021-03-03

**Authors:** Hyocheol Bae, Sunwoo Park, Changwon Yang, Gwonhwa Song, Whasun Lim

**Affiliations:** 1 Institute of Animal Molecular Biotechnology and Department of Biotechnology, College of Life Sciences and Biotechnology, Korea University, Seoul 02841, Korea; bhc7@korea.ac.kr (H.B.); sunwoojump@korea.ac.kr (S.P.); ycw117@korea.ac.kr (C.Y.); 2 Department of Food and Nutrition, College of Science and Technology, Kookmin University, Seoul 02707, Korea

**Keywords:** campesterol, ovarian cancer, cell death, ROS, mitochondria dysfunction

## Abstract

Phytosterols, which are present in a variety of foods, exhibit various physiological functions and do not have any side effects. Here, we attempted to identify functional role of campesterol in regulation of oxidative stress by leading to cell death of ovarian cancer. We investigated the effects of campesterol on cancer cell aggregation using a three-dimensional (3D) culture of human ovarian cancer cells. The effects of campesterol on apoptosis, protein expression, proliferation, the cell cycle, and the migration of these cells were determined to unravel the underlying mechanism. We also investigated whether campesterol regulates mitochondrial function, the generation of reactive oxygen species (ROS), and calcium concentrations. Our results show that campesterol activates cell death signals and cell death in human ovarian cancer cells. Excessive calcium levels and ROS production were induced by campesterol in the two selected ovarian cancer cell lines. Moreover, campesterol suppressed cell proliferation, cell cycle progression, and cell aggregation in ovarian cancer cells. Campesterol also enhanced the anticancer effects of conventional anticancer agents. The present study shows that campesterol can be used as a novel anticancer drug for human ovarian cancer.

## 1. Introduction

Phytosterols are steroids produced by plants. They are similar to cholesterol and include stanols and phytosteroids. Phytosterols have been reported to inhibit low density lipoprotein cholesterol and protect against cardiovascular disease in several studies [1]. Foods and dietary supplements containing phytosterols have been consumed by humans for decades. Phytosterol-containing functional foods have been monitored in the EU market since 2000, and little is known about its side effects [2]. Depending on their structure, phytosterols are involved in specific physiological reactions. They are the precursors of plant hormones and brassinosteroids and regulate the growth and development of plants [3,4,5]. Moreover, they affect intracellular signal transduction through the formation of specific lipid microdomains (lipid rafts) in the membrane [6,7]. In addition, diets high in phytosterols have been reported to reduce the risk of developing ovarian cancer [8]. Campesterol is a phytosterol that is found in a variety of vegetables, fruits, nuts, and seeds and is abundant in canola and corn oil [9]. Hence, campesterol is one of the most common phytosterols, along with β-sitosterol and stigmasterol. It is competitively absorbed with cholesterol through the human intestine owing to its structural similarity with cholesterol. Campesterol can regulate carrier proteins, intestinal cells, and lipid metabolism, including the synthesis and esterification of cholesterol and assembly of lipoproteins [10]. The anticancer effects of campesterol have been reported frequently in recent years. Campesterol inhibits the growth of leukemia, hepatocarcinoma [11] and prostate cancer cells [12,13]. In several studies, the consumption of campesterol (10 mg/day) was shown to reduce the risk of cancer by 13%. It was suggested that the risk of cancer could be gradually reduced in inverse proportion to the level of campesterol intake [14]. However, there are no reports that campesterol suppresses human ovarian cancer.

Among the ovarian cancer, epithelial ovarian cancer is most frequent (90%) type, and it histologically distinguished as serous (52%), endometrioid (10%), clear cell (6%), mucinous (6%), or undistinguished subtypes [15]. The incidence and mortality of ovarian cancer have continued to decline. The mortality caused by ovarian cancer has decreased by more than 30% in recent decades as a result of improved treatment [16]. Nevertheless, survival within five years after diagnosis is less than half for high-grade serous carcinoma because of rapid metastasis, difficulties in early diagnosis, and the high rate of recurrence [16]. Given that preserving functional mitochondria is a key feature of drug-resistant ovarian cancer cells, phytochemicals targeting mitochondria have the potential to increase the therapeutic response in ovarian cancer [17]. In addition, inducing ER stress is an effective way to enhance the sensitivity of existing drugs against ovarian cancer, as it induces the apoptosis of ovarian cancer cells [18].

We aimed to identify anticancer compounds from natural materials that do not have side effects. Although the anticancer effects of campesterol have been reported in several cancer types, little is known about its effect on organelles, such as mitochondria and ER, as well as the underlying mechanism by which it induces apoptosis. Moreover, there is no information on the functional role of campesterol, especially in ovarian cancer cells. In the present study, we found that campesterol induced apoptosis in two ovarian cancer cell (serous carcinoma and clear cell carcinoma) lines. It also regulated intracellular mechanisms for cell survival, including mitochondrial function and homeostasis of the ER. Moreover, campesterol promoted the expression of cell death factors and inhibited the growth of cells by inhibiting the expression of proteins related to cell growth. The production of ROS and levels of calcium were also dramatically increased by campesterol in both the cell lines. These results provide mechanistic insights into the anticancer effects of campesterol on human ovarian cancer.

## 2. Materials and Methods

### 2.1. Reagents

Campesterol (cat no: CFN92204) was purchased from ChemFaces (Wuhan, China). It was dissolved in dimethyl sulfoxide (DMSO) before the treatment of cells.

### 2.2. Cell Culture

Ovarian cancer cells (ES2; ovarian clear cell carcinoma cells and OV90; papillary serous adenocarcinoma cells) were purchased from American Type Culture Collection (ATCC; Manassas, VA, USA). Cells from both cell lines were incubated in McCoy’s 5A medium containing 10% fetal bovine albumin (FBS). The cells were grown in a carbon dioxide cell culture incubator (37 °C, 5% CO_2_). All the cells were incubated in medium lacking FBS for 24 h before treatment to assess the effects of campesterol.

### 2.3. 3D Cell Culture

The cells were cultured by hanging them on the cover of a culture dish (3 × 10^3^ cells/drop). Treatment with the vehicle or campesterol (125 µM) was performed for 72 h. The formation of cancer was assessed by visualization under a DM3000 microscope (Leica, Wetzlar, Germany). The aggregated cancer cell area was estimated using ImageJ software. The 3D structure density of aggregated cancer cells was calculated using the ReViSP software. The experiment was performed in triplicate.

### 2.4. Apoptosis Assay

Cells from both cell lines were grown and FBS starved in monolayer culture condition for 24 h. The cells were then treated with campesterol (0, 25, 62.5, and 125 µM) for 48 h and then rinsed twice with PBS to remove the treatment solution. Subsequently, the cells were trypsinized and collected by centrifugation. The collected cells were incubated with FITC Annexin V (BD Biosciences, Franklin Lakes, NJ, USA) and propidium iodide (PI) for 15 min. Fluorescence was observed using a flow cytometer (BD Bioscience). For all experiments, the results were estimated in comparison with those obtained for cells treated with the vehicle control. The experiment was performed in triplicate.

### 2.5. Western Blot Analysis

Cells from both cell lines were incubated with campesterol (0, 25, 62.5, and 125 µM) for 24 h. Western blot analysis was performed, as described previously [19]. The experiment was performed in triplicate.

### 2.6. JC-1 Staining

The cells were treated with campesterol (0, 25, 62.5, and 125 µM) in monolayer culture condition for 48 h and subsequently rinsed twice to remove the treatment solution before they were harvested. The detached cells were collected by centrifugation and rinsed twice with PBS. They were then stained with JC-1 (Sigma-Aldrich, St. Louis, MO, USA) for 20 min at 37 °C, rinsed twice with JC-1 buffer, and analyzed using a flow cytometer (BD Bioscience). The experiment was performed in triplicate.

### 2.7. ROS Production

The cells were grown and FBS starved in monolayer culture condition for 24 h. They were detached by trypsinization and collected by centrifugation; subsequently, the cells were washed twice with PBS. The collected cells were treated with 2′,7′- dichlorofluorescein diacetate (DCFH-DA) (Sigma, 10 µM) for 30 min, rinsed twice with PBS, and incubated with campesterol (0, 25, 62.5, and 125 µM) for 1 h. ROS was detected using a flow cytometer (BD Bioscience). The experiment was performed in triplicate.

### 2.8. Evaluation of Cytosolic Calcium Levels

The cells were grown and FBS starved in monolayer culture condition for 24 h. They were treated with campesterol (0, 25, 62.5, and 125 µM) for 48 hours and then rinsed twice with PBS to remove the treatment solution before they were harvested. The detached cells were collected by centrifugation, rinsed twice with PBS, and stained with fluo-4 acetoxymethyl ester (AM; 3 μM) (Invitrogen, Waltham, MA, USA) for 20 min at 37 °C. The cells were again washed twice with PBS; subsequently, fluorescence from the fluo-4 dye was detected using a flow cytometer (BD Bioscience). The results of all the experiments were compared with those of the control group. The experiment was performed in triplicate.

### 2.9. Evaluation of Mitochondrial Calcium Levels

The cells were grown and FBS starved in monolayer culture condition for 24 h. They were treated with campesterol (0, 25, 62.5, and 125 µM) for 48 h and then rinsed twice with PBS to remove the treatment solution. Subsequently, the cells were trypsinized, collected by centrifugation, and washed twice with PBS. They were then stained with rhod-2 dye for 20 min at 37 °C and rinsed twice with PBS; subsequently, fluorescence from rhod-2 was determined using a flow cytometer (BD Bioscience). The results of all the experiments were compared with those of the control group. The experiment was performed in triplicate.

### 2.10. Cell Proliferation

Cell proliferation was investigated in monolayer culture condition by ELISA using a BrdU Kit (Roche, Basel, Switzerland). The cells were grown and FBS starved for 24 h. They were treated with different concentrations of campesterol in a 96-well plate for 48 h. The cells were stained with BrdU for 2 h, and the presence of BrdU was probed by incubation with anti-BrdU-POD for 1 h 30 min. The cells were washed thrice with PBS, and color was developed via the addition of a chromogenic substrate, which was read on a microplate reader (Bio-Tek, Winooski, VA, USA). The experiment was performed in triplicate.

### 2.11. Cell Cycle

Cells were treated with campesterol in monolayer culture condition for 48 h and then rinsed twice to remove the treatment solution. They were trypsinized and collected by centrifugation and then treated with RNase A and PI for 30 min. Fluorescence from PI was determined using a flow cytometer (BD Bioscience). The results of all the experiments were compared with those of the vehicle-treated group. The experiment was performed in triplicate.

### 2.12. Migration

The cells were grown on Transwell inserts and treated with campesterol or vehicle for 12 h. They were subsequently fixed with methanol and stained with hematoxylin. The membrane was rinsed and covered with mounting medium. The migration of cells was analyzed by visualization under a DM3000 microscope (Leica). The experiment was performed in triplicate.

### 2.13. Quantitative Real Time PCR

The expression levels of the selected genes were determined by quantitative RT-PCR using SYBR green dye, as described previously [20]. The experiment was performed in triplicate.

### 2.14. Statistical Analysis

All quantitative data were subjected to least squares ANOVA using the General Linear Model procedures of the Statistical Analysis System (SAS Institute Inc., Cary, NC, USA). Western blot data were corrected for differences in sample loading using total protein data as a covariate. All tests of significance were performed using the appropriate error terms according to the expectation of the mean squares for error. A *p*-value less than or equal to 0.05 was considered significant. Data are presented as least-square means (LSMs) with SEs (*** = *p* < 0.001, ** = *p* < 0.01, and * = *p* < 0.05).

## 3. Results

### 3.1. Activation of Cell Death and Restriction of Cell Aggregation by Campesterol in Human Ovarian Cancer Cells

**Figure 1:**
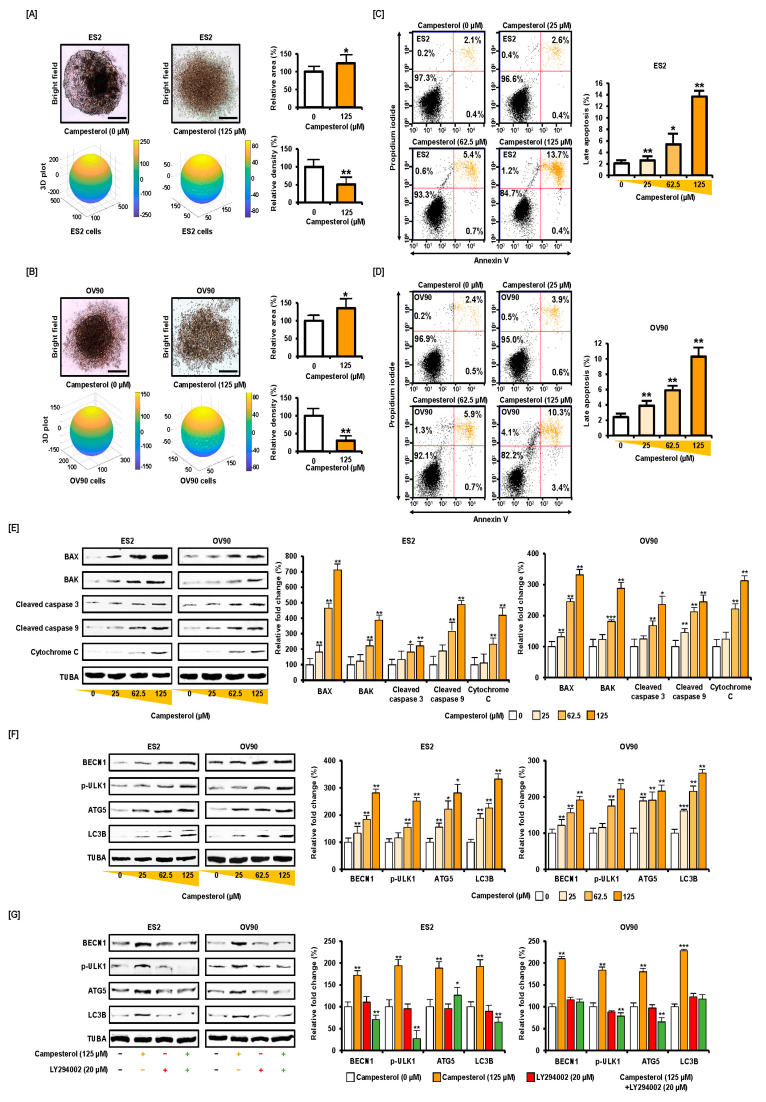
Inhibition of cancer cell aggregation and activation of cell death by campesterol in human ovarian cancer cell lines. (**A**,**B**) Comparison of aggregation in the control and campesterol-treated cells. (**C**,**D**) A cell apoptosis assay was conducted to investigate the induction of apoptosis in the cells treated with campesterol. The quadrants in the dot plot show the cells in the different phases of apoptosis. The graph shows the changes in the percentages of cells in the late apoptosis phase. (**E**,**F**) Western blot analysis showing the activation of the proapoptotic proteins and autophagy proteins upon treatment with campesterol (0, 25, 62.5, and 125 µM). (**G**) Western blot analysis of proteins from ES2 and OV90 cells treated with Campesterol, LY294002, or co-treated with both. The data represent three independent experiments. The asterisks indicate significant differences between the treated cells and control cells (*** *p* < 0.001, ** *p* < 0.01, and * *p* < 0.05).

We tested the effects of campesterol on a 3D ovarian cancer model. The hanging ovarian cancer cells (3 × 10^3^ cells/drop) were treated with the vehicle or campesterol (125 µM). The aggregated cancer cell area was estimated using ImageJ software. The 3D structure was calculated using the ReViSP software. Campesterol suppressed the aggregation of human ovarian cancer cells. The area of the aggregated ovarian cancer cells was increased to 123% and 135%, respectively, for each cell line ES2 and OV90 compared to that in the vehicle-treated controls (100%). However, the density of the ovarian cancer cells was reduced to 50% and 30%, respectively, for both the cell lines compared to that in the vehicle-treated group (100%) (Figure 1A,B). These data show that the aggregation of the ovarian cancer cells was suppressed by campesterol treatment. Next, we investigated the apoptosis induced by campesterol (0, 25, 62.5, and 125 µM) in the ovarian cancer cells using annexin V and propidium iodide (PI) staining (upper right quadrant in Figure 1C,D). In the ES2 cells, the percentage of cells in the late apoptosis phase was increased to 2.6%, 5.4%, and 13.7% upon treatment with 25, 62.5, and 125 µM of campesterol, respectively, compared with that in the vehicle-treated control (2.1%) (Figure 1C). In the OV90 cells, the cell population in the upper right quadrant was increased to 3.9%, 5.9%, and 10.3% upon treatment with 25, 62.5, and 125 µM of campesterol, respectively, compared with that in the vehicle-treated control (2.4%) (Figure 1D). The results of western blot analysis showed that campesterol activated the expression of proapoptotic proteins in both cell lines. Treatment with campesterol (0, 25, 62.5, and 125 µM) stimulated the cleavage of caspase 3 and caspase 9 in both cell lines in a dose-dependent manner. It also induced the expression of cytochrome C, BAK, and BAX in both cell lines (Figure 1E). In contrast, the expression of alpha tubulin (TUBA) remained unchanged upon treatment with campesterol in both cell lines. In addition, campesterol increased the autophagy related protein expression such as BECN1, phosphorylated (p)-ULK1, ATG5, and LC3B compared with those of TUBA in both cell lines (Figure 1F). However, LY294002, an inhibitor of several proteins required for autophagy, suppressed the activation of autophagy signals in ES2 and OV90 cells (Figure 1G).

### 3.2. Alterations in the Mitochondrial Membrane Potential (MMP) and ROS Levels by Campesterol

**Figure 2:**
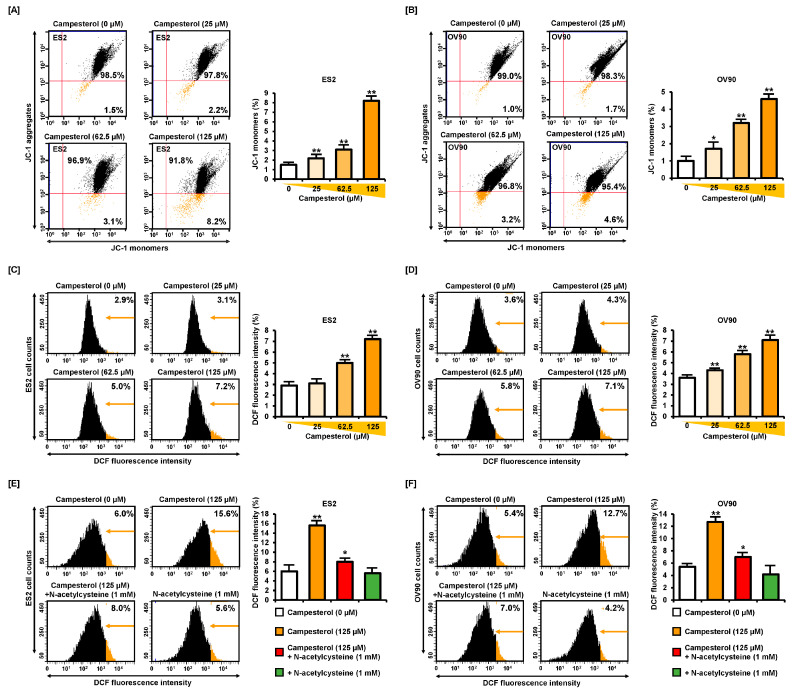
Alterations in the MMP and production of ROS by campesterol. (**A**,**B**) MMP was determined after treatment with campesterol (0, 25, 62.5, and 125 µM) using JC-1 staining. The graph shows the loss of MMP compared with that in the control. (**C**,**D**) The changes in the levels of ROS caused by treatment with campesterol were determined using the DCF dye. The histogram shows the ROS production in comparison with that in the control. (**E**,**F**) N-acetylcysteine alleviated ROS production after cotreatment with campesterol and N-acetylcysteine. DCF: dichlorofluorescein. The data represent three independent experiments. The asterisks indicate significant differences between the treated cells and control cells (** *p* < 0.01, and * *p* < 0.05)

All campesterol (0, 25, 62.5, and 125 µM) treatment altered the mitochondrial function in the ES2 and OV90 cells in monolayer culture condition. In the ES2 cells, the loss of MMP, quantified as the proportion of JC-1 monomers, was increased to 2.2%, 3.1%, and 8.2% upon treatment with 25, 62.5, and 125 µM of campesterol, respectively, compared with that in the control (1.5%) (Figure 2A). Similarly, in the OV90 cells, the proportion of JC-1 monomers was increased to 1.7%, 3.2%, and 4.6% upon treatment with 25, 62.5, and 125 µM of campesterol, respectively, compared to that in the control (1.0%) (Figure 2B). ROS was investigated in ovarian cancer cells with treatment of campesterol (0, 25, 62.5, and 125 µM) for 1 h using DCFH-DA, 10 µM staining. In the ES2 cells, the generation of ROS, quantified by dichlorofluorescein (DCF) fluorescence intensity, was increased to 3.1%, 5.0%, and 7.2% upon treatment with 25, 62.5, and 125 µM of campesterol, respectively, compared with that in the control (2.9%) (Figure 2C), whereas in the OV90 cells, it was increased to 4.3%, 5.8%, and 7.1% upon treatment with 25, 62.5, and 125 µM of campesterol, respectively, compared with that in the control (3.6%) (Figure 2D). Flow cytometry analysis was conducted after cotreatment with campesterol and N-acetylcysteine (NAC; 1 mM). Campesterol increased ROS levels in ES2 cells (15.6%) and OV90 cells (12.7%). However, NAC mitigated the increase in ROS levels in ES2 cells (8.0%) and OV90 cells (7.0%) (Figure 2E,F).

### 3.3. Upregulation of the Cellular and Mitochondrial Calcium Concentrations by Campesterol

**Figure 3:**
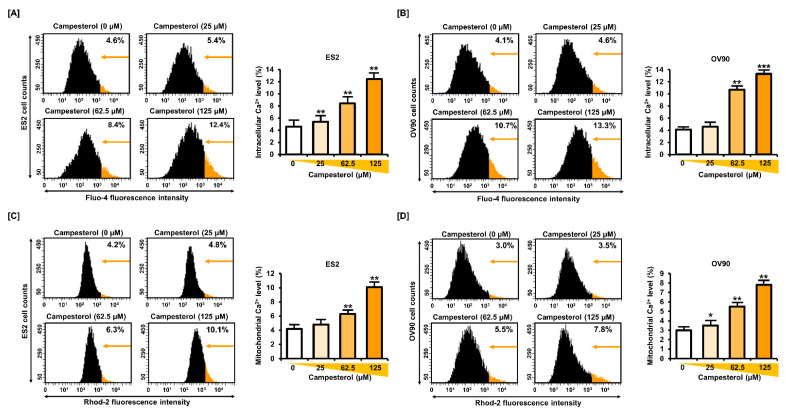
Concentrations of calcium in the cytosol and mitochondria of cells treated with campesterol. (**A**,**B**) Cytosolic levels of calcium were determined using fluo-4 fluorescence. The histogram represents the alterations in the intracellular calcium levels upon treatment of the cells with campesterol (0, 25, 62.5, and 125 µM). The graphs show the changes in the intracellular calcium levels upon treatment of the cells with campesterol compared to that in the control. (**C**,**D**) Rhod-2 fluorescence was measured to determine the changes in the concentrations of calcium in the mitochondria. The histogram represents the changes in the mitochondrial calcium concentrations upon treatment of the cells with campesterol. The graphs show the alterations in the concentrations of calcium in the mitochondria upon treatment of the cells with campesterol compared with that in the control. The data represent three independent experiments. The asterisks indicate significant differences between the treated cells and control cells (*** *p* < 0.001, ** *p* < 0.01, and * *p* < 0.05).

To determine the changes in the levels of calcium, we treated the cells with 0, 25, 62.5, and 125 µM of campesterol in monolayer culture condition. Thereafter, the calcium levels in the cytosol and mitochondria were determined using fluo-4-AM and rhod-2 fluorescence, respectively. In the ES2 cells, the cellular calcium levels, quantified by fluo-4 fluorescence intensity, were increased to 5.4%, 8.4%, and 12.4% upon treatment with 25, 62.5, and 125 µM of campesterol, respectively, compared with that in the control (4.6%) (Figure 3A), whereas they were increased to 4.6%, 10.7%, and 13.3%, respectively, compared with that in the control (4.1%) in the OV90 cells (Figure 3B). The levels of calcium in the mitochondria, quantified by rhod-2 fluorescence intensity, increased to 4.8%, 6.3%, and 10.1% in the ES2 cells (Figure 3C) upon treatment with 25, 62.5, and 125 µM of campesterol, respectively, compared with that in the control (4.2%) and to 3.5%, 5.5%, and 7.8% in the OV90 cells (Figure 3D) upon treatment with 25, 62.5, and 125 µM of campesterol, respectively, compared with that in the control (3.0%).

### 3.4. Activation of ER Stress and the ER–Mitochondrial Axis by Campesterol in the ES2 and OV90 Cells

**Figure 4:**
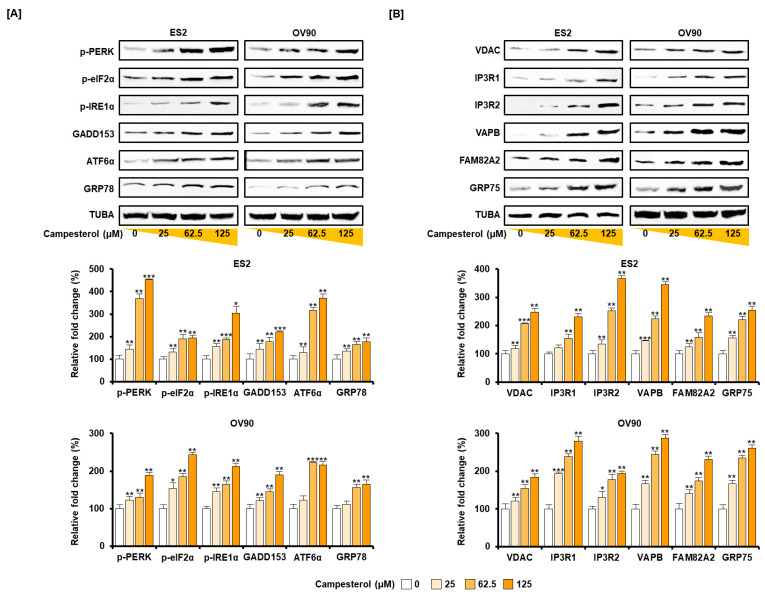
Activation of the ER-stress sensor and the ER–mitochondrial axis signals by campesterol in the ovarian cancer cells. (**A**) Western blot analysis of ER-stress proteins in the cells treated with campesterol (0, 25, 62.5, and 125 µM). (**B**) Western blot analysis of the ER–mitochondrial axis proteins in the cells treated with campesterol (0, 25, 62.5, and 125 µM). TUBA was used as a control. The graph represents the relative fold changes in the levels of proteins induced by campesterol treatment compared with that in the control (100%). The data represent three independent experiments. The asterisks indicate significant differences between the treated cells and control cells (*** *p* < 0.001, ** *p* < 0.01, and * *p* < 0.05).

Ovarian cancer cells were treated with campesterol (0, 25, 62.5, and 125 µM) for 24 h. Western blot analysis revealed the changes in ER stress and the ER–mitochondrial axis. The accumulation of UPR proteins, including p-PERK, p-eIF2α, IRE1α, GADD153, ATF6α, and GRP78, was induced by campesterol (0, 25, 62.5, and 125 µM) treatment compared with that of TUBA in both cell lines (Figure 4A). Additionally, the expression of ER–mitochondria axis proteins, including VDAC, IP3R1, IP3R2, VAPB, FAM82A2, and GRP75 were increased upon campesterol treatment compared with those of TUBA in both cell lines (Figure 4B).

### 3.5. Inhibition of Cell Proliferation and the Cell Cycle by Campesterol

**Figure 5:**
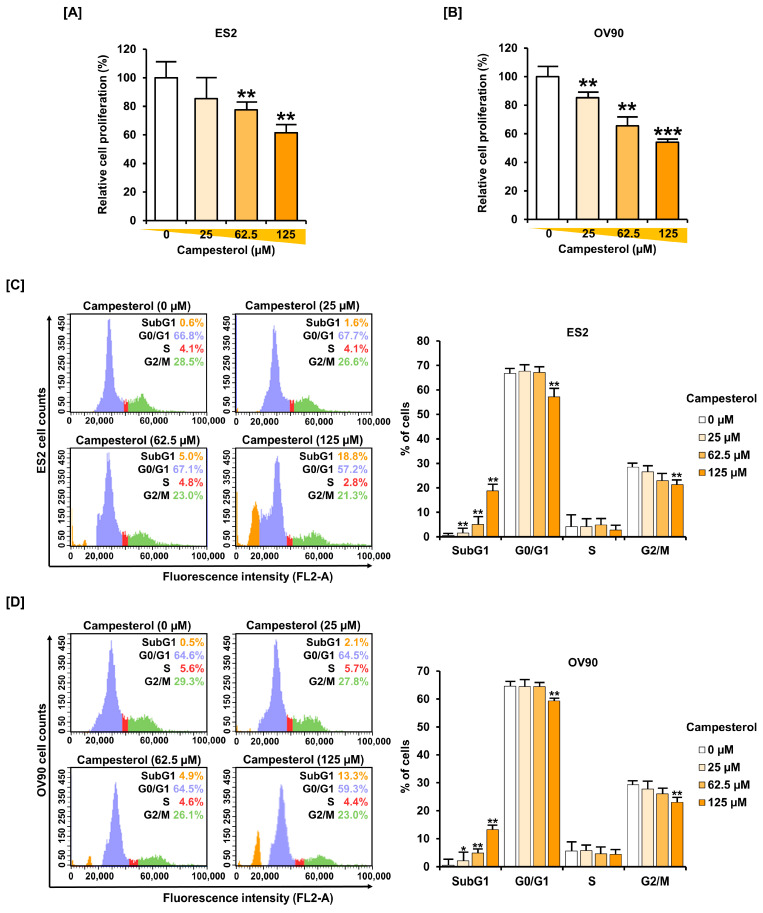
Inhibition of cell growth by campesterol in the ovarian cancer cell lines. (**A**,**B**) The cell proliferation assay in the campesterol (0, 25, 62.5, and 125 µM)-treated cells. The graphs show the percentage of cell growth compared with that of the control cells (100%). (**C**,**D**) The histogram presents the cell cycle of the campesterol (0, 25, 62.5, and 125 µM)-treated cells. The graphs represent the percentages of the campesterol (0, 25, 62.5, and 125 µM)-treated cells in the sub-G1, G0/G1, S, and G2/M phases. The data represent three independent experiments. The asterisks indicate significant differences between the treated cells and control cells (*** *p* < 0.001, ** *p* < 0.01, and * *p* < 0.05).

Cell proliferation was observed using a BrdU ELISA Kit. Treatment of campesterol (0, 25, 62.5, and 125 µM) suppressed proliferation in both cell lines. The proliferation of the ES2 cells was inhibited by up to 39.0% at 125 µM of campesterol (EC50 = 245.13 µM) (Figure 5A). The cell proliferation of the OV90 cells was reduced by up to 46.0% at 125 µM of campesterol (EC50 = 147.13 µM) (Figure 5B). In terms of cell cycle progression, ovarian cancer cells were treated with campesterol (0, 25, 62.5, and 125 µM) for 48 h and staining with RNase A and PI. The sub-G1 phase was increased by up to 18.8% at 125 µM of campesterol compared with that in the control (0.6%) in the ES2 cells (Figure 5C). Whereas, in the OV90 cells, it was increased by up to 13.3% at 125 µM of campesterol compared with that in the control cells (0.5%) (Figure 5D).

### 3.6. Regulation of Signaling by Campesterol in the Ovarian Cancer Cells

**Figure 6:**
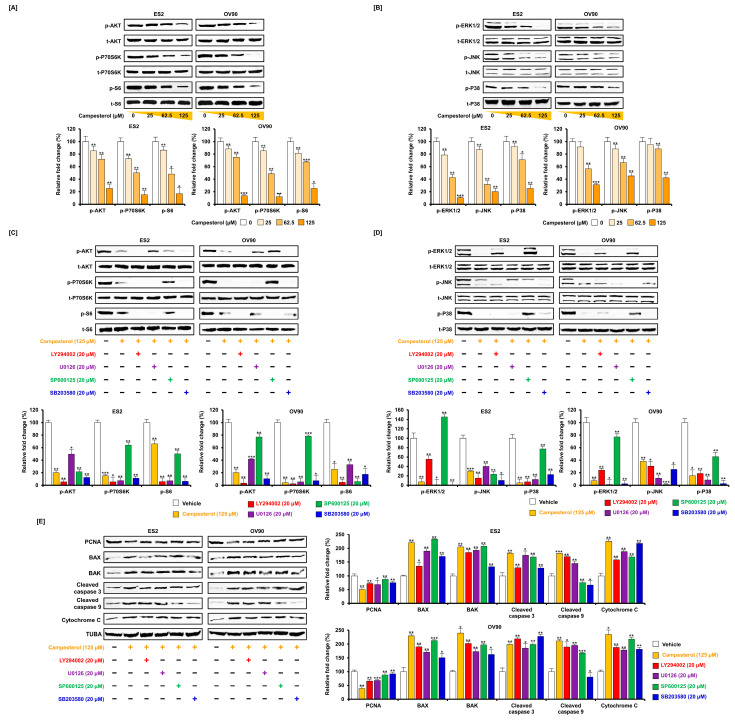
Changes in the cell growth-related and proapoptotic signals upon treatment of the cells with campesterol. (**A**,**B**) Western blots showing the changes in the PI3K/MAPK signals upon campesterol treatment in both cell lines. (**C**,**D**) Western blots showing the alterations in the PI3K/MAPK signals upon cotreatment of LY294002, U0126, SP600125 and SB203580 with campesterol in both cell lines. (**E**) Representative western blots of the levels of the proliferating and proapoptotic factors upon cotreatment with campesterol and LY294002, U0126, SP600125, or SB203580 in both cell lines. The data represent three independent experiments. The asterisks indicate significant differences between the treated cells and control cells (*** *p* < 0.001, ** *p* < 0.01, and * *p* < 0.05).

For investigate intracellular signal transduction, ovarian cancer cells were treated with campesterol (0, 25, 62.5, and 125 µM) for 48 h. The cell proliferation-related intracellular signal pathways were identified using western blot analysis. The phosphorylation of AKT, P70S6K, and S6 was decreased by campesterol 0, 25, 62.5, and 125 µM treatment compared to the levels of the respective total protein in both cell lines (Figure 6A). Additionally, the phosphorylation of ERK1/2, JNK, and p38 was inhibited in a dose-dependent manner by campesterol (0, 25, 62.5, and 125 µM) compared to the levels of the respective total protein in both cell lines (Figure 6B). Next, we determined the signal correlation in greater detail using signal cascade inhibitors, including a PI3K inhibitor (LY294002; 20 µM), ERK1/2 inhibitor (U0126; 20 µM), JNK inhibitor (SP600125; 20 µM), and P38 inhibitor (SB203580; 20 µM). The cells were treated with each inhibitor before the campesterol treatment. The phosphorylation of AKT was almost blocked by LY294002 and SB203580 in the ES2 and OV90 cells, respectively. The phosphorylation of P70S6K was inhibited by LY294002 in the ES2 cells and OV90 cells. The phosphorylation of S6 was decreased by LY294002 in the ES2 cells and inhibited by LY294002 and SP600125 in the OV90 cells (Figure 6C). The phosphorylation of ERK1/2 was inhibited by U0126 in both cell lines. The phosphorylation of JNK was completely blocked by LY294002, U0126, and SP600125 in the ES2 cells. p-JNK was repressed by U0126 in the OV90 cells. The phosphorylation of P38 was completely blocked by SB203580 in the ES2 cells and inhibited by LY294002 and SB203580 in the OV90 cells (Figure 6D). In addition, campesterol treatment resulted in a reduction of proliferating cell nuclear antigen (PCNA) levels. In addition, the combination of campesterol and each pharmacological inhibitor suppressed PCNA compared to the vehicle-treated control in both cell lines. Campesterol-induced the expression of several proapoptotic protein, which in some cases was prevented by cotreatment with the anti-apoptotic inhibitors. BAX protein levels were decreased by LY294002, U0126, and SB203580 treatment in ES2 cells, and by all inhibitors in OV90 cells. BAK protein levels were diminished by SB203580 in ES2 cells and by all inhibitors in OV90 cells. Cleaved caspase 3 levels were decreased by LY294002 and SB203580 in ES2 cells and slightly increased by LY294002 and SB203580 in OV90 cells. Cleaved caspase 9 and cytochrome c were decreased by all the inhibitors in both cell lines. For the western blot analysis, the proapoptotic protein levels were compared to those of TUBA (Figure 6E).

### 3.7. Reductions in the Migration and Expression of Angiogenic Genes in the Ovarian Cancer Cells Treated with Campesterol

**Figure 7:**
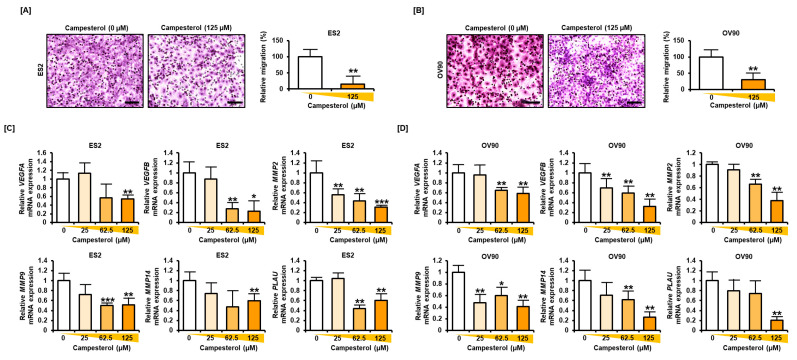
Campesterol inhibits cell migration and angiogenic gene expression. (**A**,**B**) The migration of cells was investigated using Transwell inserts. For each cell line, five non-overlapping locations were visualized. (**C**,**D**) The expression of genes involved in angiogenesis was determined by quantitative RT-PCR. Scale bar represents 100 μm. The data represent three independent experiments. The asterisks indicate significant differences between the treated cells and control cells (*** *p* < 0.001, ** *p* < 0.01, and * *p* < 0.05).

The cells were treated with campesterol (125 µM) and stained with hematoxylin. The migration of the ovarian cancer cells was decreased to 84.7% in the ES2 cells and 69.3% in the OV90 cells upon treatment with campesterol compared with that in the control cells (Figure 7A,B). Moreover, the expression levels of the genes that play key roles in migration and angiogenesis, including *vascular endothelial growth factor A* (*VEGFA*), *VEGFB*, *matrix metalloproteinase-2* (*MMP2*), *MMP9, MMP14,* and *plasminogen activator, urokinase* (*PLAU*) were decreased upon campesterol treatment in both cell lines (Figure 7C,D).

### 3.8. Enhancements in the Anticancer Effects of Existing Drugs by Campesterol

**Figure 8:**
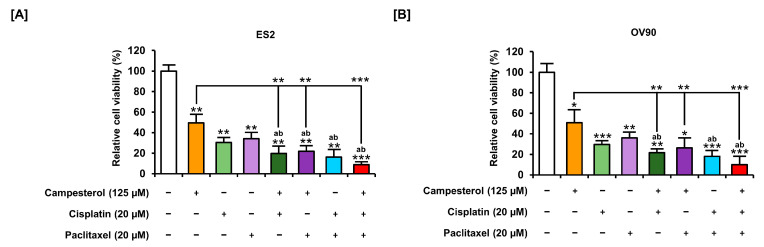
Reduced cell proliferation upon cotreatment with campesterol and existing drugs in ovarian cancer cell lines. (**A**,**B**) The proliferation of cells treated with campesterol and existing drugs was determined in relation to that of the control cells (100%). Statistical significance was also shown between the group treated with campesterol alone and the group treated with the conventional drug plus campesterol. The data represent three independent experiments. The asterisks indicate significant differences between the treated cells and control cells (*** *p* < 0.001, ** *p* < 0.01, and * *p* < 0.05). ‘a’ and ‘b’ indicate significant differences as compared to cisplatin and paclitaxel, respectively.

Cell proliferation assays were performed to determine whether campesterol has synergistic effects with existing anticancer drugs. The cells were incubated with campesterol (125 µM) alone or were co-treated with campesterol and cisplatin (20 µM) or paclitaxel (20 µM). Cell proliferation were compared with vehicle-treated control group (100%). Campesterol (125 µM) decreased the cell proliferation to half in both cell lines. Moreover, the combination treatment of campesterol and cisplatin or paclitaxel inhibited cell proliferation more than the treatment alone. Campesterol enhanced the inhibition of cell growth by cisplatin and paclitaxel in both ovarian cancer cell lines (Figure 8A,B).

## 4. Discussion

A case-control study revealed that diets high in phytosterols are associated with a reduced risk of ovarian cancer [8]. A posterior meta-analysis revealed that only campesterol was inversely related to cancer risk [14]. Campesterol induces the apoptosis of cells and inhibits cell proliferation in histiocytic lymphoma (U937 cells), liver cancer (HepG2 cells) [11], prostate cancer (PC-3 cells) [13], and breast cancer (MDA-MB-231 cells) [21]. It was also reported to repress basic fibroblast growth factor (bFGF)-induced angiogenesis through the regulation of cell proliferation and differentiation [7]. Campesterol inhibited cell proliferation through the inhibition of the liver X receptor, which is important for cell proliferation in prostate and breast cancers [12]. In addition, campesterol caused DNA damage, the activation of caspase, and cell cycle arrest at the G1 and G2/M phases in T lymphocytes (Jurkat cells), lymphoma cells (Jeko-1 cells), and glioma cells (LN22 cells) [22]. Similarly, in our study, campesterol induced late apoptosis and suppressed cell growth in both the selected ovarian cancer cell lines. In addition, campesterol increased the proportion of cells in the G1 phase of the cell cycle. These results indicate that campesterol induces apoptosis in human ovarian cancer cells. 

Campesterol induced cell apoptosis through the mitochondrial pathway; it decreases the expression of BCL-2 and BCL-xL and increased the expression of BAX, BAD, BAK, and activating caspase 3 and caspase 9 in human lung adenocarcinoma (A549) cells [23]. Campesterol was reported to reduce the growth of liver cancer (SSMC-7721) cells by inducing cell cycle arrest, apoptosis, ROS generation, the loss of MMP, a decrease in Bcl-2 expression, and the activation of caspase 3 and caspase 9 [24]. In ovarian cancer, campesterol also activated cell death signals, such as cleaved caspase 3, cleaved caspase 9, cytochrome c, BAX, and BAK. In addition, the function of mitochondrial membranes was reduced upon treatment of cells with campesterol. The depolarization of mitochondrial potential and the secretion of proapoptotic signals causes programed cell death [25]. Early stage of programmed cell death is caused by disruption of mitochondria function, which includes changes in the membrane potential, a central feature of mitochondrial health, and alterations to the oxidation–reduction potential of the mitochondria. The MMP is essential in Ca^2+^ uptake and storage, ROS generation and detoxification and, most importantly, the synthesis of ATP by oxidative phosphorylation [26]. Therefore, the membrane’s depolarization is a good indicator of mitochondrial dysfunction [27]. Autophagy is a homeostatic cellular process that removes damaged organelles during cellular stress responses [28]. Autophagy is generally not only activated in nutritional deficiencies, but is also involved in a number of physiological processes including development, differentiation, neurodegenerative diseases, infections and cancer [29]. Autophagy marker light chain 3B (LC3B) is critical for autophagy and undergo post-translational modifications during autophagy [30,31,32]. The LC3-I/LC3-II ratio increases through lipidation by an ubiquitin-like system involving Atg7 and Atg3 that allows the establishment of autophagic vesicles during autophagy [33]. Also, autophagy is regulated by phosphorylation of the Unc-51-like kinases ULK1 and ULK2 [34]. ULK activates BECN1, which forms autophagy-inducible BECN1 protein complexes [35,36]. The activated ULK and BECN1 complexes initiates autophagosome through activation of downstream autophagy components [37,38]. The ubiquitin-like conjugation system involved in autophagy requires the binding of the ubiquitin-like protein Atg5 [39]. In addition, autophagy-related gene are reported as tumor suppressor including BECN1 and Atg5 [40]. In our results, these autophagy markers were significantly increased by campesterol in both cell lines. ROS play a pivotal role in cell survival, but accumulation of ROS beyond the survival threshold leads to cell death [41]. Excessive ROS production and consequent increase in intracellular calcium concentration activates apoptosis signals, along with the destruction of mitochondria [42,43]. Therefore, an increase in ROS and calcium concentration inside the cells and the mitochondria induced by campesterol is speculated to induce the death of ovarian cancer cells.

The ER is an important organelle that is involved in the production and maturation of protein in cells. Imbalance of ER homeostasis causes ER stress and unfolded protein response (UPR). When UPR fails to restore organelle homeostasis and continues to accumulate, it triggers the cell apoptosis pathway. Evidence has reported that ER stress can play a role in the anticancer activity of various plant-derived natural compounds such as curcumin, resveratrol, green tea polyphenols, tocotrienols, and garcinia derivatives [44,45]. Cellular metabolism is closely regulated by various organelles. The ER–mitochondria axis influence energy metabolism through their structure and function being dynamically regulated by nutritional and environmental signals [46,47]. However, the over-expression of ER–mitochondria signaling cause autophagic stimulus [48]. In particular, ABT-737, a non-selective Bcl-2/Bcl-XL inhibitor can actually affect the ER–mitochondrial contact site, thereby enhancing the response to cisplatin in ovarian cancer cells [49,50,51]. Campesterol has been shown to cause ER stress through the activation of the UPR and the ER–mitochondria axis in ovarian cancer cells. In addition, campesterol inhibited cell growth-related intracellular signaling, including the PI3K/MAPK cascades that are related in ER stress, in human ovarian cancer cells. Suppressing the cellular signal pathways that are mainly used by cancer cells is a useful anticancer strategy [52]. Moreover, the intraperitoneal injection of campesterol decreased the growth of tumor nodules and increased the survival rate of breast cancer-bearing mice [53]. MAPK signaling proteins including extra cellular signal-regulated kinase (ERK), Jun kinase (JNK/SAPK) and p38 MAPK regulates cell cycle engine and cell proliferation related proteins [54]. PI3K-AKT signaling is also important for proteins controlling cellular proliferation by regulating cyclins, cyclin-dependent kinases, and cyclin-dependent kinase inhibitors in cancer cells [55]. In the present study, campesterol decreased the activity of PI3K/MAPK signaling transduction pathway. Moreover, we confirmed that campesterol actually affects the PI3K/MAPK pathway and that there is a signal correlation between the two pathways that campesterol affects. PCNA is an essential protein that contributes to several cellular processes, such as cell survival, energy metabolism, DNA replication and repair, chromatin organization, and transcription. Therefore, targeting PCNA is a promising strategy for suppressing cancer cell proliferation [56]. Our study shows that campesterol decreases the expression of PCNA in ovarian cancer cells, suggesting the potential benefits of using of campesterol as a drug against ovarian cancer.

The migration of cancer cells to surrounding tissues and blood vessels causes metastasis [57]. Therefore, inhibiting the migration of cancer cells is a good anticancer strategy. Campesterol decreased cell migration in PC-3 cells [13]. In the present study, campesterol inhibited cell migration in human ovarian cancer cells. Also, angiogenesis plays key role in the supply of nutrients to cancer cells and in their metastasis [58]. Therefore, the inhibition of angiogenesis can be useful for anticancer therapy. We observed that campesterol suppressed the expression of angiogenic and migratory genes, including *VEGFA*, *VEGFB*, *MMP2*, *MMP9*, *MMP14*, and *PLAU*, in the ES2 and OV90 cells. A recent study reported that β-sitosterol may increase the sensitivity of colorectal cancer cells to anticancer drugs [59]. However, to the best of our knowledge, there has been no report on whether phytosterols can improve the sensitivity of human ovarian cancer cells to conventional anticancer drugs. In the present study, for the first time, we established that campesterol could exert synergistic effects with anticancer drugs in cancer cells. However, we did not assess this effect on drug-resistant cell lines, an aspect that requires further study. The results of this study suggest that campesterol, like other conventional drugs, induces mitochondrial-mediated apoptosis on ovarian cancer cells, as previously reported. Several studies have suggested that the preservation of functional mitochondria is one of the key factors determining the sensitivity of ovarian cancer cells to conventional drugs. This implies that mitochondrial dysfunction caused by campesterol may contribute to increased sensitivity to cisplatin and paclitaxel in ovarian cancer cells [17,60,61]. In addition, other campesterol-mediated effects such as excessive ROS production, induction of ER stress, and changes in cellular signaling pathways may also contribute to increasing the sensitivity of ovarian cancer cells to drugs such as cisplatin or paclitaxel [18,60]. These results suggest that campesterol may be used as a novel adjuvant for the treatment of human ovarian cancers.

## 5. Conclusions

Campesterol caused cell apoptosis and activated proapoptotic signals in human ovarian cancer cell lines. The treatment of cells with campesterol impaired the mitochondrial membrane function and destroyed the calcium balance. In addition, ROS generation and the expression of ER stress-sensor proteins were increased by campesterol in a dose-dependent manner. The ER–mitochondria axis-related proteins were also activated by campesterol in both cell lines. Campesterol inhibited cell growth and cell cycle progression through the regulation of the PCNA and PI3K/MAPK signal pathways. It also inhibited the aggregation of ovarian cancer cells and enhanced the anticancer effects of cisplatin and paclitaxel in these lines. These results indicate the potential use of campesterol as a new therapeutic agent for ovarian cancer.

## Data Availability

Data is contained within the article.
